# Endophthalmitis after pars plana vitrectomy with reused single-use devices: a 13-year retrospective study

**DOI:** 10.1186/s40942-020-00274-5

**Published:** 2021-01-06

**Authors:** Sukhum Silpa-archa, Kwanchanoke Kumsiang, Janine M. Preble

**Affiliations:** 1 Faculty of Medicine, Department of Ophthalmology, College of Medicine, Rajavithi Hospital, Rangsit University, 2 Phayathai Road, Ratchathewi District, Bangkok, 10400 Thailand; 2 Department of Ophthalmology, Kresge Eye Institute, School of Medicine, Wayne State University, 4717 St. Antoine, Detroit, MI 48201 USA

**Keywords:** Endophthalmitis, Pars plana vitrectomy, Recycled, Single-use devices, 23-gauge, 25-gauge, Ethylene oxide, Hydrogen peroxide

## Abstract

**Background:**

To describe the incidence, clinical characteristics, and treatment outcomes of endophthalmitis after pars plana vitrectomy (PPV) with recycled single-use devices. The recommended sterilization process as well as safety measures are discussed.

**Methods:**

Medical charts of patients who developed endophthalmitis after PPV were retrospectively reviewed and reported in a descriptive manner. Cases undergoing PPV for preexisting endophthalmitis or open globe injury were excluded. Data collection included patient demographics, operative details, ocular findings, microbiological profiles, treatment modalities, and visual outcomes.

**Results:**

Over the past thirteen years, a total of 12,989 pars plana vitrectomy operations were included. In total, 13 eyes of 13 cases (0.10%) experienced endophthalmitis after vitrectomy. These occurred in 3 cases (0.11%) using 20-gauge vitrectomy compared to 8 cases (0.09%) using 23-gauge vitrectomy and 2 cases (0.18%) using 25-gauge vitrectomy. There were no statistically significant differences between the 20-gauge and microincisional vitrectomy surgery (MIVS) group (P = 0.64), and the 23- and 25-gauge approach (P = 0.34). Causative pathogens were positive by culture in 5 cases (45%): 3 g-positive cases, 1 g-negative case, and 1 fungus case.

**Conclusions:**

The rate of endophthalmitis in patients who underwent 23-gauge PPV was comparable to those who underwent 25-gauge PPV. With our standardized protocol for instrument sterilization, endophthalmitis rates in those undergoing PPV using recycled single-use instruments were within the range of previously published results in which vitrectomy tools were disposed of after one use.

## Background

Postoperative endophthalmitis is a rare and devastating ocular entity. Endophthalmitis can occur after any intraocular surgery. Given the advancement of vitrectomy machine technology, vitrectomy has become more accessible and commonly performed by vitreoretinal specialists. With the introduction of microincisional vitrectomy surgery (MIVS), including 25-gauge and 23-gauge transconjunctival vitrectomy systems, as opposed to conventional sutured 20-gauge vitrectomy, there was concern for a possible increase in endophthalmitis rates. However, previous meta-analysis and multicenter studies showed a similar endophthalmitis risk between 20-gauge vitrectomy and MIVS [1, 2]. There are recent studies with large cohorts of both sutured and sutureless vitrectomy that show both supporting and contradictory results for MIVS as a risk of postvitrectomy endophthalmitis (PVE) [3, 4].

As the first report of PVE in the Southeast Asian population, the present study aimed to describe the incidence, clinical characteristics, and treatment outcomes of PVE in the Thai population. A mini-review of previous studies of PVE following MIVS was also included in the study. In addition, given cost-saving measures, our institute has been reusing single-use devices for vitrectomy. Thus, the present study is the first to compare rates of endophthalmitis after pars plana vitrectomy (PPV) performed with vitrectomy tools disposed of after one use with rates of endophthalmitis after PPV that used recycled single-use vitrectomy tools. The recommended sterilization process and safety measures are discussed.

## Methods

A retrospective study was initially performed by reviewing medical charts of patients developing PVE at the Department of Ophthalmology, Rajavithi Hospital, Bangkok, Thailand from January 2005 to December 2017. The study was approved and the need for informed consent was waived by the Research Ethics Committee of Rajavithi Hospital (approval No. 029/2561). The study followed the tenets of the Declaration of Helsinki.

### Study design and data collection

Inclusion criteria consisted of patients who underwent primary or repeated PPV, and patients who underwent PPV with combined or uncombined cataract extraction. The exclusion criteria were any of the following: (1) preoperative diagnosis of endophthalmitis; (2) history of vitrectomy or intraocular surgery within 6 months; (3) history of open globe injury; (4) immunocompromised patients or patients undergoing immunosuppressive therapy. We determined that patients had endophthalmitis if hypopyon or vitritis was seen on exam or if the patient received an antibiotic intravitreal injection for presumed endophthalmitis, irrespective of microbiological culture results [5, 6]. Data collected encompassed patient demographics, operative details, ocular findings, microbiological profiles, treatment modalities, and visual outcomes. In terms of operative technique, we analyzed operative risk factors including antiseptic technique, PPV methods, utilization of intraocular tamponade, presence of complications and presence of postoperative hypotony. The visual acuity recorded at the patient’s first visit for endophthalmitis was used as the presenting visual acuity in this study.

### Surgical procedure details

The PPV instrument changed from 20-gauge PPV to MIVS (23- and 25-gauge) since 2009 and the number of cases undergoing 20-gauge vitrectomy has declined considerably. In order to prevent development of endophthalmitis, a preoperative assessment was done to ensure the patient had no periocular and/or ocular surface infection and that there was no obstruction within the lacrimal drainage system. Antibiotic eye drops were not routinely prescribed prior to PPV. We used gauze soaked with 10% povidone-iodine solution to disinfect the perioribital area. Afterwards, we diligently cleaned the periorbital area with two cotton swabs soaked with 10% povidone-iodine solution, and irrigated the conjunctival sac with 10 mL of 5% povidone-iodine solution as previously described [7]. After removal of each cannula, the sclerotomy was compressed with the forceps tip to close the scleral wound [8]. All sclerotomies were evaluated for leakage at the completion of each procedure. When prolapse of transparent vitreous through the scleral wound was identified, it was excised with a vitreous cutter and the scleral wound was closed with 1 stitch of 8-0 Vicryl suture through the conjunctiva. All patients received antibiotic eye drops and combined antibiotic/corticosteroid ointment before patching at the conclusion of the case. Antibiotic injections for prophylaxis were not utilized by surgeons at our institution. Surgeon’s preference determined which antibiotics (tobramycin vs levofloxacin) were used in the post-operative period. Corticosteroid eye drops were prescribed for 2 weeks. Postoperative follow-up was performed at day 1, 1 week, 1 month, 3 months and then as needed afterward. Postoperative hypotony was defined as an intraocular pressure reading of 7 mmHg or less observed within 1 week after surgery. Once the diagnosis of endophthalmitis was made, patients immediately underwent vitreous aspiration needle tap and/or PPV to isolate pathogens. Subsequently, patients underwent antibiotic intravitreal injection with vancomycin and ceftazidime. Systemic antibiotics were used at the surgeon’s discretion. Both culture-positive and culture-negative patients were enrolled, and after sampling the causative pathogens, antibiotic therapy was initiated. Rescue vitrectomy for endophthalmitis was at the surgeon's discretion.

### Sterilization methods for re-use of instruments

The Accurus (Alcon Laboratories, Inc., Fort Worth, TX) was used before being replaced by Stellaris PC (Bausch & Lomb, Rochester, NY, USA) in 2012. Disposable vitrectomy devices that have been reused in our setting include vitrectomy cassettes, trocar cannulas, vitreous cutters, endoilluminators, intraocular forceps, laser probes, and diathermy probes. The recycled items are discarded after being utilized 3 times, used in infected eyes, used on HIV-infected patients, or at the surgeon’s discretion. All reused devices are cleaned with enzymatic detergent in order to remove bulk biomaterial. Enzymatic detergent was passed through the lumen of the vitreous cutter tubing for 1 min before rinsing with sterile water. All devices except for vitrectomy cassettes and endodiathermy probes are placed in the ultrasonic cleaner. The optimal condition for ultrasonic processing is set at 40 °C for 10 min. The inside and outside of vitrectomy cassettes are then thoroughly cleaned with mild soap detergent and rinsed with soap-free sterile water. To finalize the sterilization process, all reused devices except for endodiathermy probes are then dried and packed before being sterilized by Steri-Vac^™^ sterilizers using 100% ethylene oxide (EO) as per standard protocol. The EO sterilization process in our hospital is regularly validated with class V EO indicator strips and EO biological indicators. After being cleaned with enzymatic detergent, endodiathermy probes are placed in the STERRAD^®^ 100S sterilizer. The STERRAD^®^ Chemical Indicator Strip is regularly used to verify exposure to vaporized hydrogen peroxide.

To distinguish reused materials from new materials, reused materials were labelled with a permanent marker at the end of the operation. These markings allowed us to determine the number of times instruments were reused. Once instruments were used a third time, they were discarded.

Patients were given the option to undergo PPV with a new vitrectomy pack at an additional cost or a recyclable vitrectomy pack at no additional cost. In addition to the vitrectomy pack (including vitrectomy cassette, trocar cannula, vitreous cutter and endoilluminator), other instruments were randomly selected to be reused. At least one resterilized device was used in every single operation. Therefore, a surgeon might use a resterilized vitrectomy cassette, new intraocular forceps, and a resterilized laser probe in a particular case. The degree of instrument reuse (e.g., first, second,…) was not recorded in patients' records.

### Statistical analysis

Snellen visual acuity values determined for the diseased eye were transformed to the logarithm of the minimum angle of resolution (logMAR). This scale was converted to logMAR values: counting fingers, 2.00; hand motion, 2.30; light perception, 2.60; and no light perception, 2.90 [9, 10]. Statistical analysis was performed using the IBM SPSS Statistics for Windows, Version 20.0 (Armonk, NY: IBM Corp; 2011). Descriptive analyses were utilized in order to describe demographic characteristics, pre-operative assessment, surgical procedures, and treatment outcome. A univariate analysis by chi-square test or Fisher’s exact test was performed to indicate the significance of categorical variables. The independent *t *test was used to compare continuous variables. All statistical tests were two-tailed and significance was defined as *P* < 0.05.

## Results

### Demographic and operative data

Over a 13-year study period, a total of 12,989 PPVs were performed in our center and 13 eyes (0.10%; 1 in 999 cases) of PVE were identified in 13 patients. Since 2009, we have changed from sutured vitrectomy (20-gauge) to MIVS including 23-gauge and 25-gauge sutureless vitrectomy. For all operations, 20-gauge, 23-gauge and 25-gauge PPV made up 2,798 (21.5%), 9,102 (70%) and 1,089 (8.4%) of the operations. Postvitrectomy endophthalmitis occurred in 3 cases (0.11%) with 20-gauge vitrectomy compared to 8 cases (0.09%) with 23-gauge vitrectomy and 2 cases (0.18%) with 25-gauge vitrectomy. There were no statistically significant differences between the 20-gauge and MIVS group (P = 0.64), and the 23- and 25-gauge approach (P = 0.34). Of the total 13 PVE cases, the median age at presentation was 68 years (49–83). There were four men and nine women. Table 1 shows the detailed demographic and clinical summary of cases with postvitrectomy endophthalmitis. All patients were immunocompetent and not using immunosuppressive treatment. Preoperative risk factors including diabetes 23% (3/13) and hypertension 38% (5/13) were present. The indications for vitrectomy were lens-related complications (39%, 5/13), epiretinal membrane (38%, 5/13) and diabetic tractional retinal detachment (23%, 3/13). Two patients had blunt ocular injury with traumatic lens dislocation indicated for surgery. The median time of operation was 55 (range, 25–90) minutes. The intraocular tamponade used was 54% (7/13) balanced salt solution, 38% (5/13) air, and 8% (1/13) silicone oil. No intraocular fluid leakage was detected after surgery and there was no hypotony. Sixty-two percent of patients (8/13) with PVE had a universal coverage health scheme. The median duration between vitrectomy and presentation of endophthalmitis was 5 days (1–13). None of the PVE cases were febrile at initial presentation. Hypopyon was found in 54% (7/13) of cases. Fundus examination was completely obscured in 77% (10/13) of cases. The median follow-up period was 6 months (1–156). Median best-corrected visual acuity (BCVA) before vitrectomy and at initial presentation of endophthalmitis was 2.00 and 2.30 logMAR.

**Table 1:** Detailed demographic and clinical summary of cases with postvitrectomy endophthalmitis

Case	Sex/Age/Eye	Medical history	Indication of vitrectomy	Type of surgery (gauge)	IOL placement/ Endotamponade used	Causative organisms	Intravitreal agents (number of injections)	Systemic treatment	Rescue PPV	Complications	Presenting BCVA (Log MAR)	Final BCVA (Log MAR)
1	F/74/R	None	EMM	20	N/Fluid	ND	VC (1), CT (1)	Levofloxacin	Y	None	2.60	0.20
2	F/49/R	DM	Diabetic TRD	20	N/Air	MRSA	VC (1), CT (1)	Ciprofloxacin	N	RRD	2.60	2.90
3	F/63/L	HT	EMM	20	N/Fluid	NG	VC (1), CT (1)	Gatifloxacin	Y	None	2.30	0.50
4	M/71/L	HT, DLP	EMM	23	N/Fluid	*Enterococcus faecium*	VC (3), CT (3)	Ciprofloxacin	Y	None	2.30	0.50
5	F/63/R	HT	EMM	23	N/Fluid	NG	VC (1), CT (1)	Ciprofloxacin	N	None	2.00	0.80
6	F/69/L	Parkinson disease, Down syndrome	IOL dislocation	23	Y/fluid	NG	VC (1), CT (1)	-	Y	None	2.30	0.70
7	F/81/L	HT	IOL dislocation	23	Y/fluid	*Proteus mirabilis*	VC (2), CT (2)	-	Y	None	2.60	2.90
8	F/83/L	DM	Diabetic TRD	23	N/Air	*Staphylococcus lugdunensis*	VC (4), CT (1), CD (3)	Ciprofloxacin	Y	Elevated IOP	2.60	2.30
9	F/57/L	DM, HT	Diabetic TRD	23	Y/silicone oil	*Aspergillus flavus*	VC (1), CT (1)	Ciprofloxacin	N	Elevated IOP	2.60	2.90
10	F/68/R	None	EMM	23	N/Fluid	NG	VC (1), CT (1)	Ciprofloxacin	Y	RRD	2.30	2.90
11	M/56/L	None	Traumatic lens dislocation	23	Y/air	ND	VC (3), CT (3)	Ciprofloxacin	N	None	2.00	2.90
12	M/58/L	None	Traumatic lens dislocation	25	Y/air	NG	VC (3), CT (3)	Ciprofloxacin	N	None	0.70	0.80
13	M/78/R	None	Intra-operative lens dislocation	25	Y/air	NG	VC (3), CT (3)	Ciprofloxacin	Y	Elevated IOP	2.30	0.50

*M* male, *F* female, *L* left, *R* right, *DM* diabetes mellitus, *HT* hypertension, *DLP* dyslipidemia, *EMM* epimacular membrane, *IOL* intraocular lens, *TRD* tractional retinal detachment, *Y* yes, *N* no, *ND* not done, *NG* no growth, *MRSA* methicillin-resistant *Staphylococcus aureus*, *PPV* pars plana vitrectomy, *VC* vancomycin, *CT* ceftazidime, *CD* clindamycin, *RRD* rhegmatogenous retinal detachment, *IOP* intraocular pressure

### Causative microorganisms of endophthalmitis

Intraocular fluid specimens were obtained for investigation in 85% (11/13). A positive culture was demonstrated in 45% (5/13) (Table 1). Gram-positive bacteria were the most commonly identified organisms (60%, 3/5). Methicillin-resistant *Staphylococcus aureus* was isolated in one patient who underwent 20-gauge PPV for severe diabetic tractional retinal detachment. There was only one patient (no. 9) with fungal endophthalmitis caused by *Aspergillus flavus.* This patient had poorly controlled diabetes for 7 years. He presented with BCVA of light perception in the left eye before undergoing PPV with silicone oil tamponade for a severe combined tractional and rhegmatogenous retinal detachment. Postoperative BCVA was not improved on day 1 and remained stable when endophthalmitis developed. His vision became no light perception when the positive culture of *Aspergillus flavus* was reported at 1 month. He denied further treatment and, eventually, the eye became phthisical at 6 months.

### Treatment and visual outcome

The overall median BCVA at 1 month after endophthalmitis treatment was improved from initial presentation at 2.3 logMAR (20/4000) to 0.90 logMAR (20/160). Sixty-two percent of patients (8/13) experienced visual improvement after treatment and 23% (3/13) had BCVA better than or equal to 0.5 logMAR (20/60) at 1 month after treatment. At 1-month follow-up, median BCVA of patients with PVE following 20-, 23- and 25-gauge PPV was 1.0 (20/200), 2.5 (20/6000) and 0.4 logMAR (20/50). There was no significant difference amongst median BCVA at 1-month follow-up between the 20-gauge and MIVS group (P = 0.81). Patients with PVE following 25-gauge vitrectomy demonstrated better visual outcome than those following 23-gauge vitrectomy (P = 0.04) at 1-month follow-up. All patients with PVE were hospitalized and received intravitreal antibiotic injections of vancomycin and ceftazidime. Systemic oral antibiotics were employed in 85% (11/13). All systemic antibiotics administered were oral fluoroquinolones. Rescue vitrectomy for endophthalmitis was performed in 62% (8/13). Of these, intraocular tamponade by silicone oil was performed in only one case due to rhegmatogenous retinal detachment. None of the patients underwent evisceration or enucleation. Post-treatment complications included elevated intraocular pressure (23%, 3/13) and rhegmatogenous retinal detachment (15%, 2/13). All cases with elevated intraocular pressure were well-controlled on antiglaucoma eye drops. Fifteen percent of patients developed phthisis bulbi.

## Discussion

Our work presented cases of PVE from a large cohort of patients. From a systematic literature review, most large studies of PVE following MIVS presented different types of gauges as a whole, without delineating them into 23-gauge, 25-gauge or 27-gauge PPV [3, 4, 11–13]. These reports could be confounded and, instead, should be clarified separately. As such, the current study represents a group of PVE in a large cohort of 23-gauge PPV. Nowadays, most vitreoretinal surgeons use transconjunctival sutureless vitrectomy or MIVS (23-, 25-, or 27-gauge) for their practice instead of the sutured 20-gauge. Therefore, the discussion primarily emphasizes MIVS. The overall incidence of PVE in all types of gauge and only MIVS reported from previous studies was 0.02–0.84% and 0.01–0.84%. For 23-gauge and 25-gauge, the incidence of PVE in the former was 0.03–0.08%, while the incidence in the latter was 0.02–0.84% [2–4, 11, 12, 14–23]. The incidences of PVE demonstrated by the most recent systematic review and meta-analysis was 0.03% after 23-gauge, and 0.11% after 25-gauge [23]. Though the incidence rates from our study are higher than the pooled incidence from the meta-analysis, they were within the range of published values. In addition, after the meta-analysis was published, the most recent report of PVE after 23-gauge PPV by Lin et al. [19] found that the incidence rate of PVE after 23-gauge was 0.08% (3/3,979) which is similar to our data. The higher incidence of PVE after 25-gauge PPV compared with 23-gauge was consistent with our data. Also, previous studies of PVE in the early era of MIVS found 25-gauge PPV had a statistically significant higher incidence of PVE compared with 20-gauge PPV [17, 21]. Possible causes are related to the changed properties of MIVS like 25-gauge and included incision, mechanism, and fluidics as follows: (1) unsutured sclerotomy incisions with a higher tendency of introduction of normal flora and hypotony after operations; (2) minimal PPV with higher amounts of retained vitreous at the completion of the procedure; (3) distinctly lower flow rate through the 25-gauge infusion as compared with the 20-gauge cannula [21]. As the technique evolved, only the incision seemed to be reasonable enough to be attentive and manageable. The Microsurgical Safety Task Force by an expert panel recommended guidelines for minimizing the risk of endophthalmitis associated with MIVS. The guideline included an application of 10% povidone-iodine, conjunctival displacement, angled or tapered incision, intraocular tamponade the eye with air, avoidance of vitreous incarceration, wound assessment, and postoperative subconjunctival and topical antibiotics [24]. We followed these suggestions especially the use of 10% povidone-iodine, angled incision, and careful wound assessment for vitreous incarceration and integrity. The reduction in incidence of PVE is distinct for the 25-gauge approach in which surgeons tended to do a straight 90° incision of the trocar into the eye due to the smaller diameter of gauge. After the adoption of angling of scleral incision, we have not seen any PVE cases following a 25-gauge approach since 2013. However, it should be interpreted with caution due to the small number of PPV cases performed. Only a few studies [2, 25, 26] in the direct comparison of 23- and 25-gauge MIVS also demonstrated no significant differences between the two groups, which was consistent with our study (P = 0.34).

In fact, the rate of endophthalmitis following PPV in the modern MIVS is similar to one following phacoemulsification [6, 27, 28]. As previously reported, PVE is usually limited to postoperative hypotony secondary to leakage of intraocular fluid from the sclerotomies [2, 29]. The present study did not find postoperative hypotony in patients with PVE. Likewise, postoperative hypotony conditions in most previous studies were reported as no cases [2, 4, 17, 20–22] and very few cases [2, 3] (Tables 2, 3).

**Table 2:** Incidence and characteristics of acute-onset endophthalmitis after 23-gauge pars plana vitrectomy

Study (year)	Present study (2019)	Lin et al. [19]	Xiang-yu et al. [15]	Lihteh et al. [26]	Oshima et al. [2]	Parolini et al. [18]
Period of study (number of years)	2005–2017 (13)	2011–2014 (4)	2002–2012 (11)	2005–2009 (5)	2003–2008 (6)	2003–2008 (6)
No. of cases per patients (incidence rate)	8/9102 (0.09)	3/3979 (0.08)	0/632 (0)	3/10,845 (0.03)	2/6660 (0.03)	0/943 (0)
No. of cases with postoperative hypotony (%)	0	NA	–	NA	0	–
Intraoperative injections (number of infected eyes receiving injections)	0	NA	–	SC antibiotics^a^ (2/3)	NA	–
Median onset of endophthalmitis (days)	5 (1–6)	2 (1–5)	–	3 (2–3)	1.5 (1–2)	–
No. of cases with culture-positive (%)	4/8 (50)	2/3 (67)	–	2/3 (67)	2/2 (100)	–
Causative Microorganisms (%)	SL, EF, PM, AF	SE, ES	–	SE, SA	ES, MSSA	–

*NA* not available, *SC* subconjunctival, *SL* *Staphylococcus lugdunensis*, *PM* *Proteus mirabilis*, *AF* *Aspergillus flavus*, *SE* *Staphylococcus epidermidis*, *ES* *Enterococcus faecalis*, *SA* *Staphylococcus aureus*, *MSSA* methicillin-sensitive *Staphylococcus aureus*

^a^Types of antibiotics were not given

**Table 3:** Incidence and characteristics of acute-onset endophthalmitis after 25-gauge pars plana vitrectomy

Study (year)	Present study (2019)	Lihteh et al. [26]	Oshima et al. [2]	Chen et al. [22]	Scott et al. [17]	Shimada et al. [20]	Kunimoto et al. [21]
Period of study (number of years)	2005–2017 (13)	2005–2009 (5)	2003–2008 (6)	2002–2006 (5)	2005–2006 (2)	2004–2007 (4)	2004–2006 (3)
No. of cases per patients (incidence rate)	2/1089 (0.18)	1/4717 (0.02)	6/8238 (0.07)	1/431 (0.23)	11/1307 (0.84)	1/3343 (0.03)	7/3103 (0.23)
No. of cases with postoperative hypotony (%)	0	NA	1/6 (17)	0	NA	0	0
Intraoperative injections (number of infected eyes receiving injections)	0	0	NA	SC DEX (1/1)	SC cefazolin (9/11)	SC tobramycin and DEX (1/1)	Gentamicin in infusion fluid (7/7)
Median onset of endophthalmitis (days)	9 (5–13)	5	2 (1–3)	7	3 (1–15)	1	2 (2–10)
No. of cases with culture-positive (%)	0/2 (0)	0/1 (0)	3/6 (50)	0/1 (0)	7/11 (63)	1/1 (100)	NA
Causative Microorganisms (%)	–	–	MRSE (67)	–	CNS (86)	ES	NA

*NA* not available, *SC* subconjunctival, *DEX* dexamethasone, *ES* *Enterococcus faecalis*, *CNS* coagulase-negative *staphylococcus*, *MRSE* methicillin-resistant *Staphylococcus epidermis*

We have developed a standardized protocol that ensures sterility of ophthalmic surgical instruments and disposable tubing for years. According to the aforementioned protocol, one may be skeptical of the risk of toxic anterior segment segment syndrome (TASS) and contamination. Regarding the risk of TASS, Leder et al. conducted an investigation of enzymatic detergents as a potential cause of TASS in rabbit eyes, and their results do not support residual enzymatic detergents on surgical instruments as a cause for TASS [30, 31]. Use of high-temperature sterilizers to sterilize phaco handpieces has been well studied [32]. However, most of the vitrectomy instruments can be damaged by such heat sterilization. Therefore, to preserve the structural integrity of the instruments, we have sterilized our instruments with low-temperature sterility methods (Steri-Vac^™^ sterilizers using 100% EO) and STERRAD^®^ 100S sterilizer using hydrogen peroxide gas plasma). Both EO and hydrogen peroxide gas plasma sterilization have been used in ophthalmology [33–35] and proven for effectiveness even when challenged by a lumen as small as 1 mm [35, 36]. Ethylene oxide sterilization is a cost-effective, low-temperature process that uses ethylene oxide gas to reduce the level of infectious agents [37]. It penetrates extremely well into lumens and channels and also into the device materials themselves. Therefore, there are no defined lumen restrictions [38]. Calogero et al. evaluated the reactivity of EO contaminants to ophthalmic viscosurgical devices (OVD) in eyes of a rabbit model and found that EO exposure of an OVD was not associated with inflammation [39]. However, time is needed to remove the residual ethylene oxide from the devices before they can be used for patient care. Sen et al. reported a cluster of central retinal artery occlusion (CRAO) following cataract surgery with posterior capsule rupture, which could be associated with vitrectomy probes and tubing sterilized by ethylene oxide with inadequate aeration time of 12 h. To solve this problem, they increased the aeration time to 48 h and did not encounter any further CRAO cases after cataract surgery [35]. The aeration time used in our setting was 72 h which is much longer than the recommended safety period. We have never faced any CRAO cases after cataract surgery. Although low-temperature hydrogen peroxide gas plasma is superior to ethylene oxide sterilization in terms of much shorter processing times and harmless by-products [40], instructions for use showed that it may be limited by small lumen penetration (less than 0.7 mm). As a result, our lumened instruments were sterilized by EO sterilization after pre-cleaning with an enzymatic detergent solution and ultrasonic processing while endodiathermy probes were cleaned with STERRAD^®^ 100S sterilizer.

In regards to the risk of contamination, we reviewed the study performed by Pinto et al. evaluating microbial growth on reused single-use vitrectomy probes in Brazil healthcare practice. Without a standardized cleaning protocol, the study received used vitrectomy probes with two extensions donated from four different institutions. All samples were inoculated in culture medium and incubated at 37 °C for 14 days. The results demonstrated microbial growth on 57/979 (5.8%) sample units. They concluded that the reprocessing of single-use vitrectomy probes is not recommended [41]. However, we perceive the difference of the study settings and want to highlight two points. First, the samples sent to investigate in such study were used multiple times (2–10 times) while, in our protocol, the reused items are discarded after being utilized  ≥ 3 times. Second, their sterilizing protocol did not include passing enzymatic detergent through the lumen of tubing, rinsing with sterile water, and cleaning with an ultrasonic cleaner, while our standardized process encompasses all of these important procedures. As a result of the difference in the nature of the study, we are confident that our data from real-life practice has proved safety of the sterilization protocol for vitrectomy instruments.

Tables 2 and 3 demonstrate a comparison of the incidence and characteristics of acute-onset endophthalmitis after MIVS, and show that the rate of PVE following MIVS is within the range of published values in the setting that disposed of vitrectomy tools after one use. This proves that proper cleaning and sterilization can effectively and economically prevent microbial contamination. However, appropriate reuse requires preserving the structural integrity of the instrument. We thus limit usage to up to three times or at the surgeon’s discretion if any reduced tool performance is noted. We found this guideline can maintain the performance of vitrectomy devices and does not prolong operating time. Furthermore, the recycling policy has reduced large amounts of medical waste generated by disposable devices. Nevertheless, as rising concerns about medicolegal issues, we do recommend including the recycle policy in the surgical consent form. Patients must be given the option to go for PPV either with new instrument packs and additional charges or recyclable packs that are free of charge.

Unlike phacoemulsification, the benefit of intraoperative use of antibiotic prophylaxis was not evident for PPV [42–44]. Though previously recommended by The Microsurgical Safety Task Force, the later systematic review by Govetto and colleagues revealed the probability of benefit for using subconjunctival antibiotics was only 0.81, and estimates were uncertain [45]. Weiss et al. recently conducted a large comparative case series of transconjunctival 23-, 25-, and 27-gauge PPV over a 5-year period and revealed that prophylactic subconjunctival antibiotics were not associated with a significantly reduced rate of PVE [13]. Further studies are required to prove safety and efficacy of other routes of antibiotic prophylaxis. For example, intracameral or intravitreal injection of moxifloxacin at the conclusion of vitrectomy for prevention of PVE.

The final visual outcomes of PVE, for both 20-gauge and MIVS were varied and poor [3, 4, 19, 21]. Though our study demonstrated a significant difference of BCVA at 1 month after endophthalmitis treatment between the 23- and 25-gauge groups (P = 0.04), we believe, this is not of clinical importance because of the small number of cases. The result must be kept in perspective and requires validation.

The limitations of this study include retrospective data collection, small number of cases and a single institution was used for our results database. Pathogen isolation from the vitrectomy instrument to prove sterility was also missing.

## Conlcusion

Our study demonstrated comparable incidence rates of PVE between 23- and 25-gauge vitrectomy. Unprecedentedly, with our standardized protocol for instrument sterilization, it also disclosed that the endophthalmitis rates after PPV with recycled single-use instruments were within the range of published results in the setting that disposed of vitrectomy tools after one use. The recommended and crucial guidelines for prevention of PVE include the use of 10% povidone-iodine, angled incision, and careful wound assessment for vitreous incarceration and integrity. Further studies are needed to prove the safety and efficacy of other routes of antibiotic prophylaxis.

## Data Availability

Not applicable.

## Abbreviations

MIVS: Microincisional vitrectomy surgery
PVE: Postvitrectomy endophthalmitis
PPV: Pars plana vitrectomy
EO: Ethylene oxide
logMAR: Logarithm of the minimum angle of resolution
TASS: Toxic anterior segment segment syndrome
OVD: Ophthalmic viscosurgical device
CRAO: Central retinal artery occlusion
